# "The Hospital" Nursing Mirror

**Published:** 1899-10-28

**Authors:** 


"The Hospital" Nursing Mirror.

Being the Nursing Section of "The Hospital."

[Contributions for this Section of "The Hospital" should be addressed to the Editor, The Hospital, 28 & 29, Southampton Street, Strand, London, W.C., and should have the word "Nursing" plainly written in left-hand top corner of the envelope.]

### Notes on News from the Nursing World.

### A "PRINCESS CHRISTIAN" HOSPITAL SHIP.

An excellent suggestion was made at a meeting at Windsor on Saturday on behalf of the fund of the Central British Red Cross Committee for Nursing the Sick and Wounded during the War. After various subscriptions, including the magnificent sum of £5,000 from Mr. W. W. Astor, had been announced, it was proposed by the Dean of Windsor that, "With a view to identifying the fund raised in Windsor and district, this meeting respectfully asks her Royal Highness Princess Christian to permit her name to be given either to a hospital train, rest station, or ship set apart for nursing the sick and wounded; and, if permission be given, to request the Committee of the Central Red Cross Committee to apply some portion of the fund to support whichever scheme they consider the most practicable." The Princess Christian will not, we are sure, refuse the permission sought, and in a very short time we hope to learn that a perfectly equipped vessel bearing the name of the "Princess Christian" has started for South Africa, with a staff of the Princess Christian's own nurses.

### WAR NURSES.

The absorbing interest that follows the movements of our troops in the Transvaal naturally extends to the nurses who are being sent out to tend the sick and wounded. Five Sisters of the Army Nursing Service are already at Maritzburg, and eleven of the Army Nursing Reserve have been selected from the volunteers for duty in South Africa. Attached to No. I. Hospital Train are: Miss M. Falcon (No. of badge, 39), of Edinburgh; and Miss E. Sainsbury (46), of the Society of Chartered Nurses. To No. II. Hospital Train Miss M. L. T. Babb (72); and Miss R. L. Woollcombe (69), of the Nurses' Co-operation. Miss M. Lowe (57), of the Society of Chartered Nurses, has been appointed to No. I. General Hospital; Miss P. Watson (96), independent nurse, to No. II. General Hospital; Miss E. M. Whiteman (55), of the Society of Chartered Nurses, to No. III. General Hospital; and Miss E. Laughton (71), of the Nurses' Co-operation, to No. IV. General Hospital. Numerous ships have been offered to the Central Red Cross Committee, and attached to the vessel selected will be three nurses of the Army Nursing Reserve—Miss A. B. Brebner (97) and Miss H. Hogarth (34), of the Princess Christian's Nursing Home at Windsor; and Miss A. Spooner (58), who has been on the staff of the Royal Free Hospital since April 15th, 1890. Miss Spooner has been granted unpaid leave for a year by the hospital authorities in order that she may undertake this work. As to uniforms, the cloak is of thick navy cloth, turned up with a hem six inches deep at the bottom of fine scarlet cashmere, and the hoods are lined with the same. The dress is of fine navy serge, and the washing dresses of light bluish linen. The caps and capes are the same as those of the Army Sisters, and are made from patterns supplied by the military authorities. The bonnet is plain blue, closely fitting, without veil, and tied with white ribbon strings. Eton collars and regulation cuffs complete the costume. Each nurse is supplied with eight strapped white linen aprons, but the solar hats will be bought in South Africa.

### THE TRAINING OF THE WAR NURSES.

Here are some details of the training of the Army Nursing Reserve selected for service. Miss Sainsbury was trained at Wirral Child's Hospital, Birkenhead, and was subsequently staff nurse in the same institution. She next had a year's training at the Royal Berks Hospital, Reading, and was then assistant matron for nearly a year, afterwards serving as sister at the South Devon and Exeter Hospital, Plymouth, for several years. Miss Babb was trained at Nightingale Home, St. Thomas's Hospital, and was an extra staff nurse until October, 1895. Miss Woollcombe was trained at St. Bartholomew's, had nearly two years' experience at the training institution in connection with the hospital, and before joining the Nurses' Co-operation was engaged for a year in private nursing. Miss Lowe was trained at the Cottage Hospital, Higher Wycombe, and the Royal Free Hospital, Gray's Inn Road, where she served as staff nurse and sister until October, 1898. Miss Watson was also trained at the Royal Free Hospital. Miss Whiteman was trained at the Horton Infirmary, Banbury, and St. Bartholomew's Hospital, Rochester, where she was staff nurse for a year. Subsequently she was charge nurse at Rotherham Hospital, and head nurse at the General Hospital, Great Yarmouth. She holds a medal for her services in the typhoid epidemic at Maidstone. Miss Laughton was trained at the Royal Southern Hospital, where she was staff nurse for upwards of ten years. Prior to joining the Nurses' Co-operation she was attached to the Home Hospitals' Association. Miss Spooner was trained at the Evelina Hospital, and the Royal Free Hospital, with which she has since been connected—first as staff nurse, then as private nurse, and lastly as sister.

### NURSES AND THE PRESIDENT OF THE ROYAL COLLEGE OF SURGEONS.

The nurses who are being sent to South Africa will be among those who rejoice that Sir William MacCormac is patriotically going to the front. In the capacity of consulting surgeon to the Royal Army Medical Corps he will have control of the nurses as well as of civilian surgeons, and this is a guarantee that all the arrangements will be of the most satisfactory kind. Sir William is also personally extremely popular among the members of the nursing profession. It is pleasant to hear that the Red Cross workers in South Africa, whether they are attached to the British or the Boer forces, speak warmly of the good feeling which prevails among the wounded of both sides. There is no doubt that the wounded Boers will derive as much advantage from the skill of the President of the College of Surgeons as our own soldiers who need his services.

### A NURSES' FUND FOR THE SICK AND WOUNDED.

THERE is a movement for the formation of a nurses' fund for the benefit of the Red Cross Society, and to assist the fund for the soldiers' wives and children. Several matrons of the largest hospitals have been asked to organise it, and a correspondent appeals to us to make the idea known through our columns to all sections of the nursing world. Individual nurses can only, as a rule, afford to contribute a very small sum; but it need hardly be pointed out that a large number of very small sums means a respectable total.

### THE NURSES AT HERBERT HOSPITAL, WOOLWICH.

MISS L. W. TULLOCH has taken over the duties of acting superintendent of nurses at Herbert Hospital, Woolwich, in the place of Miss A. Garriock, who has gone to South Africa; and Sisters W. Massey, E. W. Gray, and A. Teasdale, of the Army Nursing Reserve, have been sent to Woolwich to fill the vacancies caused by the departure of three nursing sisters to the seat of the war. Miss Tulloch was trained at the Crumpsall Infirmary, Manchester, was private nurse at the Sarah Acland Home, Oxford, from 1881 to 1884, and at the Hollond Institute, Nice, from 1885 to 1887. In the latter year she entered the Army Nursing Service, and was at Netley for more than a year. She was in Egypt from 1888 to 1894, and, besides being stationed at Cairo and Alexandria, was on active service through the Nile campaign of 1889. In 1897 she was the recipient of the Royal Red Cross. The three sisters selected for duty in South Africa sailed in the hospital ship No. 10 last week.

### ON THE WAY TO PLAGUE DUTY.

ONE of the nurses on board the S.S. "Oceana," on her way to plague duty, sends us an account of her experiences. At Paris, having a couple of hours to wait for the train to Marseilles, a party of four nurses went to a restaurant for dinner.

"Upon our return to the platform," she says, "we found the train crowded and no seats to be had. There I was, running up and down the platform with a huge bundle of rugs under my arm, a sewing machine in one hand, a hat-box in another. At last we managed to get hold of Cook's agent —there were at least thirty people, French, German, English, Italian, trying to hang on to his coat-tails, all jabbering as hard as they could in their own tongues, and he trying in vain to answer and satisfy them all. After a time he took pity on us, and went to the comptroller and got him to put on a special train, consisting of two carriages, a vanguard, and engine. Six of us managed to get into one compartment, but being such a short train it rocked so much that some of the nurses were quite sick. By the time we reached the station at Marseilles we were a sorry-looking party. When we arrived at Port Said, after breakfast at the Continental, one of the doctors had the courage and the kindness of heart to escort seven of us (all mounted on donkeys) through the Arab village. A native dragoman also accompanied us. That ride was the most amusing one I have ever had. Some of our party could not ride, and when they looked as if they were going to fall off a couple of donkey-boys would run up on either side and hold them on. One lost her hat, another all her hairpins. Later on we paid the English hospital a visit. The matron kindly took us all over the building and into her sitting-room, and gave us such delicious tea. She said she was delighted to have us, as visitors seldom came to see her."

### THE QUESTION OF NUNS AND NURSING AT GALWAY.

THE managers of the Galway General Hospital recently selected for the position of matron Sister Mary Bernard Ryan, of the Mercy Community, with the result that a number of persons in Galway sent a memorial to the Irish Local Government Board protesting against the appointment of nuns to the charge of the hospital, and subsequently the Local Government Board refused to sanction the choice of Miss Ryan. Since then the managers of the hospital have met and decided to refuse to nominate any other person, and there the matter stands.

### THE END OF THE LOWESTOFT HOSPITAL TROUBLE.

IT is with much satisfaction that we learn peace has been restored among the officials at the Lowestoft Hospital, and that consequently the change in the *personnel*, which a few days ago seemed to be indispensable, need not be insisted upon. At the adjourned meeting of governors the report on the respective charges of the matron and Dr. Walker, to which we alluded last week, was presented and considered. The findings in this document were unanimously arrived at, and without going into details—which it is no longer necessary to discuss—it may be said that they did much to prepare the way for the happy result subsequently arrived at. Dr. Walker was so satisfied with them that he withdrew his resignation, and signified a wish that the matron should not offer the apology which was suggested by the committee; while Miss Howes voluntarily and in gracious language intimated, through one of the members, that she regretted the use of an expression to which Dr. Walker took exception, and had no intention of being uncivil. The episode may now, accordingly, be regarded as closed; and it may be hoped that in future the matron and Dr. Walker will work in harmony in the interests of the excellent institution with which they are associated.

### VISITORS TO FEVER HOSPITALS.

LAST week the members of the Northallerton Rural Council were surprised to receive a request from the head nurse of the hospital asking the council to give her authority to refuse visitors admission to the patients under her charge. It transpired that on the previous Sunday one patient in the convalescent stage had no less than eleven visitors, and that the efforts of the superintending nurse to interfere with them had been set at defiance. The council at once complied with the request, and in future no visitors will be permitted to enter the fever hospital at Northallerton unless a patient is *in extremis*. It is, perhaps, not so curious that people should care to encounter the serious risks of visiting patients suffering from infectious diseases as it is that they should voluntarily by their action increase the peril of others. The rule of excluding visitors from fever hospitals cannot be too rigidly maintained both in Yorkshire and elsewhere.

### WORKING MEN AND NURSING AT STOCKPORT.

IT is interesting and encouraging to observe that the Stockport Trades and Labour Council have decided to take steps to raise a sum which will show that the workpeople of Stockport appreciate the services of the staff of the Stockport Nursing Association. At a recent meeting of the council the matter was discussed at some length, and the good work done by the nursing association was warmly acknowledged. Happily, in this case, compliments are to be supplemented by cash. The nursing association has also just held its eighth annual meeting, and, according to the report of the committee, 6,513 visits were paid in the 12 months to 285 patients. Dr. Bale, who testified from his own experience as to the excellent work that was done in a very quiet and unostentatious manner, mentioned that upwards of £12 had been contributed as "thank-offerings," and added that he did not think that this would have been possible out of the very small amounts unless the efforts of the nurses were really appreciated.

### A REAL GRIEVANCE AT THE LIVERPOOL WORKHOUSE INFIRMARY.

It appears that while the Workhouse Committee of the Liverpool City Council have been engaged, as we described last week, in ventilating a trifling dispute, they have been neglecting much weightier matters. The mother of a nurse who broke down from overwork states that, while there are nearly 100 nurses in the infirmary at Brownlow Hill, there "is work, and hard work, for 200." In these circumstances it is, as this lady urges, certainly most desirable that the committee should at once turn their attention to the nurses already on the matron's staff instead of devoting it to those outside, and "ascertain how many hours the nurses work, how many wards and patients they have under their individual charge, and how many girls have broken down under the strain." With regard to the latter point, we can scarcely believe that anything approaching to habitual breakdowns can have escaped the observation of the committee.

### NURSING IN WORKHOUSES.

Among the reports of the general inspectors published in the Annual Report of the Local Government Board which has just been issued, we notice that of Mr. Fleming, inspector for Hants, Wilts, and Dorset, in which he discusses at some length the question of nursing in workhouses, and points out the difficulty of inducing women with hospital training to accept appointments which are by no means well paid, and which have little to relieve the dull monotony of feeding and washing old people going through a slow process of natural decay. Mr. Fleming seems to think, however, that good nurses would be more easily procured if the guardians took the trouble to make their lives pleasanter and brighter. In this he is no doubt quite right; but there are other causes besides the mere dulness of the work and the surroundings which tend to keep many of the best nurses from accepting Poor Law service. The matron is a great difficulty in many of the smaller workhouses.

### DISPENSARY WORK ON THE NORTH-WEST FRONTIER OF INDIA.

The dispensary work amongst the women in India is interesting, if only on account of the great variety of cases. A nurse, who has spent ten years in medical mission work, sends us some graphic details respecting the patients: "Many come several miles walking, others on bullocks, donkeys, or carried on beds. One patient is a young Hindu girl-mother, scarcely more than a child herself; she has come for an abscess to be dressed, and is very grateful for all that has been done. Then there is a Purdah lady, with several friends to explain her case; this they take a long time to do, hardly allowing her to answer a single question herself. She squats on the floor completely covered except one eye, till after persuasion from lady doctor and permission from, perhaps, her mother-in-law, she unveils her face, and under pressure shows her tongue, and allows her pulse to be felt. A third patient is a poor wee Mohammedan baby girl; her ears are in a shocking state, with 20 or more small rings in each. The mother on being asked to remove the rings flatly refuses, and takes the tiny sufferer away, thinking very little of the doctor who cannot treat on her lines. Lastly comes a hill man with his fine wife for an operation, the wife being in a very dirty state in her black robes. Our doctor, feeling she could not undertake the case thus, told them to come the next day clean. But the husband was so anxious to get it over that he took his wife outside, changed clothes with her, and reappeared." Our correspondent adds that from 80 to 100 persons of all ages collect daily in the waiting-room, and that on the whole they are wonderfully patient.

### THE SCARCITY OF WORKHOUSE NURSES.

The difficulty experienced by boards of guardians of small unions in obtaining fully-trained nurses for their infirmaries is becoming acute in some parts of England. One board in East Anglia, after advertising in two different papers for qualified nurses to fill two vacant posts, received only three applications, one of which was withdrawn before they could be submitted to the Board for consideration, leaving them no choice but that of selecting the only two candidates who applied. Among the objections raised by nurses from a large training school is the lack of acute cases, and, consequently, experience, in small union infirmaries. The whole tone of the latter, it is also urged, is considerably lower than in larger institutions where there are resident medical officers; while the probability of an occasional operation is no inducement when it has to be done, as is frequently the case, in a very makeshift, slipshod manner. It can scarcely be a source of wonder that young nurses with their future before them do not choose this kind of work.

### SHORT ITEMS.

On Saturday afternoon the Princess Christian paid a visit to the Windsor Albert Institute for the purpose of distributing medallions and certificates to the successful candidates of the local centre of the St. John Ambulance Association. The awards were 79 in number.—Miss I. A. G. Kinahan, Nursing Sister at the Guards' Hospital, in Rochester Row, has been directed to leave for South Africa on Saturday.—An attempt was made by a member of the Driffield Board of Guardians to prevent the appointment of a new head nurse, on the ground that there are only 15 patients in the workhouse infirmary. The under nurse is not certificated, and Lady Margaret Bickersteth and other members insisted that a smaller number of patients needed skilful nursing as much as a larger one. This view was eventually adopted.—It is proposed to form a nursing association at Cefn Mawr, and the movement finds a good deal of favour in the locality. But the promoters, who have obtained £28 in subscriptions, will do well to adhere to their resolution not to start until they have increased the fund to £80.

### Lectures to Surgical Nurses.

By H. A. LATIMER, M.D. (Dunelm), M.R.C.S. of Eng., L.S.A. of London, Consulting Surgeon, Swansea Hospital; Past President of the Swansea Medical Society; Lecturer and Examiner of the St. John Ambulance Association, &c.

### INFLAMMATION OF JOINTS—LOOSE BODIES IN JOINTS AND MODERN METHODS OF REMOVING THEM CONTRASTED WITH PAST ONES.

I NOW arrive at the consideration of disease as it affects joints. Owing to the complex arrangements, and the great liability to injury of these parts, they form a large part of the work which falls to the hands of Surgeons and Nurses. When you recall the fact that a joint is made up of bones, cartilages, ligaments, synovial membranes, muscles, and overlying skin, you will readily appreciate its tendency to undergo derangement at one or more of these anatomical points; for, if we set aside for the moment the factor of accidental disturbances, we know that constitutional diseases, such as rheumatism, tubercle, and the acute infectious fevers, are particularly prone to attack these structures.

The technical term for an inflamed joint is arthritis, and to this term is added a prefix to denote the variety or cause of the inflammation from which the part is suffering. Thus we speak of a gouty, rheumatic, or tubercular arthritis, and if the disease has been by injury we say it is a traumatic arthritis.

A curious feature about these inflammations of joints is that they are apt to arise in a very insidious manner from old and neglected injuries which have been unnoticed on account of their apparent insignificance. A child, for instance, has sustained a blow, or a sprain of a knee, and the injury has been so little complained of that it has passed out of notice. Later on, maybe, he has an attack of scarlatina, and then his knee begins to swell and to give pain, and an arthritis declares itself. It is just this kind of case which I introduce to emphasise the teaching of a former lecture in this series of lessons to you, when I pointed out that among your other duties the one of careful observations of symptoms is of great importance, [for I need not tell you that it will be a great help to patient] and to doctor alike if, through your instrumentality, an early recognition of symptoms is effected.

It is not necessary that I should detail the various symptoms and results of inflammation of joints. Suffice it to say that they lead to much suffering when the disease is of an acute nature, and that the general health is apt to be much impaired by the oft-time necessary limitation of exercise which is enforced for the perfect rest of the diseased part. In this last-mentioned way many people have dated general ill-health to alteration of habits made by prolonged rest for the cure of sprained joints. Of late years new methods of treatment have been devised by which diseased joints can be kept at rest while the patient is able to go about for exercise; and you will see many children walking with crutches and with Thomas's hip-joint splints who, in the old days, would have been confined to bed for many consecutive months.

The essential feature in all treatments of these cases during their curative stages is rest. When they have assumed aspects which necessitate operative measures being taken by the surgeon we see how immensely we have advanced in this science since the introduction of the antiseptic system. An acute arthritis in the pre-antiseptic days was so serious an affair, and the general fever induced by it was of so grave a character, that the limb in which it occurred was often amputated hastily to save the patient's life by removing the prime cause of the constitutional disturbance from which he was suffering. Nowadays joints are freely opened, diseased parts in their interior are removed, and they are drained or otherwise surgically treated with impunity, and very many joints are now saved which in the old days would have been sacrificed in order that life itself might be preserved. The surgery of joints thus well merits the appellation of "conservative"—the highest aim one can have in matters of this sort. There is one especial variety of arthritis to which I may well draw your attention in illustration of the advances which have been made in our art. I allude to that which is caused by loose bodies in the joints—loose "cartilages" as they are often called. It may happen that a person suddenly finds himself unable to move his knee (for, in the majority of cases, it is here that the mischief I am speaking of is apt to declare itself); the joint may be arrested half-way between a bent and straightened position, and there is an inability to move it in any way. Together with this fixation the sufferer experiences a sharp, sickening pain, and some swelling at the joint quickly follows on these symptoms. It is plain, in such a case, that something has got in between the articular surfaces, and this "something" is generally a loose or semi-attached gristle-like substance.. In the pre-antiseptic days it was customary to deal with such cases by a very roundabout method; for it was extremely dangerous to open a joint, as the part was very liable to take on an intensely active and dangerous inflammation when air and the micro-organisms which are conveyed by it were admitted to its interior. The most successful plan then in vogue was to manipulate the joint and to try to bring the loose cartilage, which was causing the attacks of pain and swelling, near the surface. If this could be done successfully a very fine, narrow-bladed knife was introduced under the skin, at some short distance from the foreign body, and when its point had reached the latter the ligament or capsule of the joint under which it lay was pierced with its point, and the body was squeezed out of the joint, through the opening, until it lay under the skin, in the track made by the knife. This latter was then withdrawn and the whole wound was allowed to heal. When healing of the skin and of the deeper wound in the capsule was completed, and the interior of the joint was protected from the admission of air, that part under which the cartilage now lay was cut down upon and it was removed. This kind of surgery was called "subcutaneous," and we still see it practised when tendons are divided for the cure of club-foot or for the freeing of contracted ligaments. You will easily see how difficult it was to get rid of a loose body in a joint by such a method as I have been describing, and how next to impossible it was when a variety and number of such bodies existed. Now we freely open the joint, examine its interior, and remove as many of these substances as we can find there; and, if done with due care and regard for antisepticism, the operation is a very successful one. In one case I removed no less than 15 loose cartilages from one knee-joint, with the happiest results—a thing which could hardly have been done in the old days.

You may ask me what these bodies are and how they manage to get where they are found. Sometimes they are portions of the cartilages of the joint which have been broken off during some violent exertion; oftener they are detached fringes of synovial membrane which have become thickened under inflammation of that structure, and have separated from the main body. If not actually free in the joint they are apt to behave as if they were, as they are often attached to the parts from whence they came by long and narrow stalks, which allow of a considerable range of movement in them.

### Nursing Kaffirs in Cape Colony.

By a Nurse in South Africa.

At a time when all matters connected with South Africa are exceptionally interesting, the experiences of a nurse who spent nearly two and a half years in a hospital in Cape Colony, originally intended solely for the reception of natives from the surrounding districts, where a population of many thousands consists almost entirely of Kaffirs, will be particularly acceptable to our readers. Our correspondent starts by dissenting from the view that the male Kaffir is a difficult person to manage when in hospital, that he is frequently violent, and that he objects to take his medicine and to submitting to discipline. Such is not her experience. She continues :—

Our hospital, with its tents for isolated cases, can accommodate at least from 50 to 70 natives—men, women, and children. Between 200 and 300 are received in the course of the year; there are also a large number of out-patients who come daily for relief. Many Kaffirs travel hundreds of miles for treatment. The heathen, or "red blankets," as they are usually called, are a quiet, simple race of people, the men being cleaner and nicer in their habits, and altogether easier to manage than the women, who are generally great talkers and extremely idle, fond of sitting about smoking all day. Some of these huge giants are modest and docile, and delighted with the gift of a bun or a few sweets. They can bear without flinching the most acute pain, and make excellent surgical cases. Taking considerable interest in the dressing of their wounds, &c., they will watch carefully every turn in bandaging, and thus quickly acquire a fair knowledge of the art of which many feel rather proud.

Kaffirs are very quick at languages ; a few weeks' residence in hospital, with just a little instruction from a fellow-patient, will enable a man to speak enough English to make it possible for him to talk to the nurses, to whom he is almost always respectful in as far as he knows how, usually addressing them by a name which in Kaffir means literally "the chief's daughter," and is the highest title of respect that can be given to a woman. The word "please," or its equivalent, is not found in the Kaffir vocabulary. A native trying to express himself in English invariably says, "You must give me," &c. He will say "Thank you" with a broad grin upon his good-natured face. It is by no means unusual for Kaffirs, even children, to be able to speak Cape Dutch, English, and sometimes a little German.

Enteric patients, as a rule, do well if they come into hospital in the early stages of the disease, for the Kaffir does not find it irksome to have to lie still, and the amasi, or sour milk, which is the diet usually prescribed, is a favourite article of diet with him. He cheerfully gives himself into the doctor's hands to be treated as he deems best.

Both men and women are fond of medicine, and it is often a grievance when, as a patient becomes convalescent, the medical superintendent stops the mixture. To see other patients getting medicine and he none, is a thing a Kaffir cannot stand. Upon one occasion a nurse in charge of the native wards, which were very full of bad cases, found a difficulty, owing to the smallness of the nursing staff and the limited number of native servants, to get some of the cleaning done. Those convalescent patients who are able to do so are expected to assist in the cleaning of the utensils if desired. The nurse accordingly handed a Kaffir a tea-kettle, requesting him to polish it ; he refused. The need was pressing, and a happy thought struck her, and, turning to him, she said, "If you do not clean this kettle, I will not give you any medicine." In a short time the man returned with the kettle, nicely polished ; and received his reward—a dose of cough mixture, composed principally of camphor.

Kaffirs have good memories and a keen sense of justice. They usually submit to discipline cheerfully enough when those placed over them are kind as well as firm—but they resent being bullied. If spoken to harshly and treated unkindly they will sometimes become violent, but as a rule they quietly submit, although they neither forget nor forgive such conduct. A little patience, combined with kindness and courage, will frequently enable a nurse to manage, unaided, either very delirious patients, lunatics, or those under the influence of alcohol, when it might be dangerous for one of the Kaffir ward boys to approach.

Many Kaffirs suffer from loathsome native diseases ; since the introduction of European clothing cases of phthisis are becoming common among them.

Heathen Kaffirs are in many ways preferable as patients to their civilised brethren, who often quickly imitate the vices of the white man without attempting to acquire any of his virtues. A little education makes a native very insolent sometimes, but this rarely happens in hospital. The well educated Kaffirs are generally most courteous and obliging to Europeans, especially to nurses, and grateful for any little kindness or attention shown them.

### Across the Seas.

### A NOTE FROM CALIFORNIA.

### With Florence Nightingale in the Crimea.

Writing under date of October 2nd Miss Helen Sexton, a trained nurse whose lot is cast in the town of Tulare, California, says: "Reading the article by the matron of an Australian Hospital in the 'Nursing Mirror' of September 16th makes me think that it may interest some of your readers to hear that in this little town where I am nursing there is a dear old lady, widow of one of our English soldiers, named Wilson, who assisted Miss Nightingale in nursing the sick and wounded soldiers in Scutari. I often run in to see her to hear her talk about the 'good old times' in the Crimea, and, like your correspondent in Australia, I seem to glean far more from the old lady's vivid descriptions than I have ever done from books written on the subject. With her (and, of course, with my British self) there are no soldiers like British soldiers, no nurses like British nurses, and it goes without saying, no women like our Queen and 'our dear' Miss Nightingale. Moreover, the glories of a fur boa, and silk cape, and some things given by the latter to her for her and her 'little Billy,' from her description must have outrivalled any such garments in London or Paris. Her glowing accounts invariably finish up with 'If I could just catch a sight of dear Miss Nightingale before I die I'd give a year off my life.' Mrs. Wilson is older than Miss Nightingale by some years I fancy. I often take her one of my English papers and read extracts to her, leaving the paper in her possession, which she treasures as if it were worth its weight in gold, till the next replaces it. There must be some magnetic attraction in them, for the old lady has cataract on both eyes, and can hardly see a foot ahead of her. An old coloured battle picture from an ancient *Graphic* is treasured beyond price, though she can only dimly discern the principal figures. One of the doctors I work for, an American Army surgeon, is an amused listener, and likes to tell of one interview with her, when she described the way the shots and shells flew round about and in their camp. He said 'I guess that scared you?' The old lady sprang up indignantly, and answered with spirit (she is Irish), 'Scared me? No, surr; I was a British soldier's wife, and what should scare me when all round me were Britishers? No, surr, British soldiers' wives don't scare; they've nothing to scare for. Didn't I just tell you they were all round the camp, and our Miss Nightingale inside everywhere. But, then (apologetically), one can't expect for the likes of you to understand, seeing that you've never seen one of our regiments!'"

### Nursing in the Fever Hospitals.

By a Trained Charge Nurse.

If the opinions expressed in controversy now going on in these pages can be taken as fairly representative, there is evidently something deplorably faulty about a system which cannot maintain order and concord between its several grades of nurses. During the four years which have elapsed since the authorities formulated the stipulation that, to be eligible for the post of charge nurse, a candidate must possess a three years' certificate of general training from some recognised training-school, they have had ample cause for seriously reviewing its practical working, and opportunity to decide the questions—"Is the system answering?" and "If not, why not?" That a constant change of staff is inconvenient to the administration of a hospital no one can doubt, and it must be a source of considerable anxiety to the matron and medical staff. Except in one notable instance (rendered notorious by the boycott), the board hospitals are well staffed, that is to say, when the hindrances incidental to the filling of vacancies allow of the full complement. When this is otherwise, added to the matron's extra work of correspondence and interviewing is the difficult problem of stationing her staff well to meet the various exigencies; and added to the charge nurse's worries is a disheartening constant change of assistants, which is not conducive to interest on the part of assistants—nurses doing a few hours' duty in this ward, and a few days in that.

That the scheme of the Board is not answering I think no officer will deny. It would be interesting to hear the opinion of some matron or ex-matron of the Board on the cause of failure and the possible remedy. My own opinion, and that of many of my colleagues, is that the difficulty lies in the need of some incentive to excellence, and the remedy is the reconstruction of the hospitals into organised training schools on the principle of those which have made the nursing profession what it is to-day.

So long as the tie which binds its staff to a hospital is a purely mercenary one, or the material one of longer hours of ease and exceptional comforts in its home life; and so long as its matron is compelled, however unwillingly, to maintain her staff by promoting junior nurses who have been repeatedly reported as incorrigibly lazy and hopelessly incompetent, the authority of heads of wards will remain a figure of speech only, and its ranks will be shunned or speedily deserted by well qualified nurses who recognise the futility of mere individual effort.

The duties of charge nurse, broadly outlined by the Norfolk House authorities, are capable of modification or expansion, according to the methods of each individual matron, or the caprices of a more or less despotic night superintendent. Under even the best conditions the post is an impossible one. The head of a ward should have an undeniable prestige which, working side by side with her assistants at staff duty, without necessary time to order and control the requirements of her ward, she cannot possess. If the sister of a ward or block were in possession of an undivided kingdom, then the women who know how to govern wisely would enter and would remain, and their subordinates would respect as well as obey them. Thus a feeling of mutual goodwill would be inaugurated.

The inferiority of the assistant staff is less of class than of competence, more of ignorance as to what is, and should be, expected of them, than want of adaptability. The influences of their environment tend to crush the ardour with which they first entered upon their work. How is it possible to expect the same interest and skill which is displayed by a body of well-drilled probationers from a band of assistant nurses who have never been instructed in the rudiments of the drill? Give them the privileges of a probationary course, with a recognised certificate in view, and, although it be only a fever certificate, the authorities will find themselves with far better material from which to choose their heads of wards than the present staff of untrained nurses, whose promotion to those posts the authorities are at present compelled to go outside their rule to sanction.

If the publicity given by the "Nursing Mirror" to the unsatisfactory conditions of work in the London fever hospitals is instrumental in bringing about a radical reform, many thanks will be due to it from its heavily-handicapped matrons, as well as from the ward workers themselves.

### Appointments.

Cottage Hospital, Bushey Heath.—Miss Georgiana Hopper has been appointed Matron. She was trained at St. Mary's Hospital, London, and Addenbrooke's Hospital, Cambridge. Subsequently, she has been staff nurse at St. Mary's Hospital; charge nurse at the Mineral Water Hospital, Bath; superintendent for seven years of the Nursing Home, Worthing; superintendent of district nurses, Worthing; sister in charge of ophthalmic wards, and afterwards sister in charge of accident wards, East Suffolk and Ipswich Hospital.

Stratford-upon-Avon Joint Infectious Diseases Hospital.—Miss Helen A. Amos has been appointed Matron. She was trained at Northampton General Infirmary, and has since been, in succession, assistant and charge nurse under the Metropolitan Asylums Board; charge nurse at the City Hospital, Lodge Road, Birmingham; charge nurse at the City Hospital, Little Bromwich; and sister of the diphtheria block, Plaistow Hospital.

Plymouth Royal Eye Infirmary.—Miss Ada F. Wilshere has been appointed Matron. She was trained, and has had ten years' experience, at the Bristol Eye Hospital.

### Minor Appointments.

Brook Hospital, Shooter's Hill.—Miss Nancy H. Thorpe has been appointed Night Superintendent. She was trained for two years at Croydon Infirmary, and received further training for five years at Charing Cross and Metropolitan Hospitals in connection with St. John's House, Norfolk Street, Strand. Subsequently, she was charge nurse for three years at the North-Eastern Hospital, Tottenham, and for the last three years she has held the post of charge nurse at the hospital of which she has now become night superintendent.

Guildford Workhouse Infirmary.—Miss Edith Hughes has been appointed Superintendent Nurse. She was trained at Mill Road Infirmary, Liverpool, and has since been charge nurse in the same institution.

West Norfolk and Lynn Hospital.—Miss Mary Cross has been appointed Charge Nurse. She was trained at and remained at the Norfolk and Norwich Hospital for twelve years.

Colchester Hospital.—Miss Ethel Garney has been appointed Sister. She was trained at the Seamen's Hospital, Greenwich, and the Women's Hospital, Soho.

### Death in Our Ranks.

We regret to learn from the superintendent of the Institution for Nurses at Lincoln of the death of Nurse Lilian Burrows from typhoid fever.

### Echoes from the Outside World.

### AN OPEN LETTER TO A HOSPITAL NURSE.

What a dear woman the Queen is! Could anything have been more touching than the message which she sent to her people through Lord Lansdowne, the Secretary of State for War? Her words, "My heart bleeds for these dreadful losses," are echoed by nearly every woman in the land, whether she has friends at the seat of war or not. Also the little suggestion at the end, that, however much Lord Lansdowne might try to convey the Queen's "warmest, heartfelt sympathy with the near relatives of the fallen and wounded," and her "admiration of the conduct of those they have lost," his efforts would fail to express the depth of her Majesty's sorrow, was very telling. It is especially at times like these, when the whole nation is stirred, that the deeds of the Royal Family intensify our affection for them. Thus we read of the Prince of Wales at Waterloo Station bidding good-bye to the departing regiments; the Duke of Connaught at Southampton leading a cheer for the Guards; the Princess of Wales asking for funds for the wives and children of the absent soldiers; the Princess Christian pleading for the Red Cross Society; and the Duke of Cambridge, as President of the Patriotic Fund, asking the Lord Mayor to open a special fund to help the widows and orphans of those who must inevitably fall in the war. The direct or indirect result of all these appeals is that, practically, four funds have been opened at the Mansion House—one for the Patriotic Fund, called (1) Widows and Orphans; a second for the sick and wounded whilst under treatment by the British Red Cross Society, called (2) Sick and Wounded; a third for those soldiers disabled by wounds (for their benefit after they leave the service), to be administered by Lloyds Patriotic Fund, called (3) Disabled Soldiers; and a fourth for the wives and children of the soldiers, who in many cases are left almost penniless when the husband goes away, to be taken charge of by the Soldiers and Sailors' Families Association, and called (4) Wives and Children. (The "Transvaal War Fund" is, of course, quite distinct). But in all probability some special scheme for nurses will be forthcoming, so that any money you may wish to contribute may be formed into a distinct offering from the nurses of Great Britain, and not simply be added as an item to the general total, which, I hope, will be such a grand one that it will surprise even the givers themselves.

---

Saturday was a memorable day. It was the anniversary of the Battle of Trafalgar, and the Navy League had spent a week and much valuable money—the flowers alone cost £1,500—in decorating the Nelson Column, which was illuminated by the electric light in the evening. Unfortunately, the fog came and entirely did away with the beauty of the decorations. The base alone was visible to the many spectators, the tall column appearing as if cut off quite short, so completely was it hidden from view. But nothing apparently could damp the patriotic ardour of the Londoners. As early as five o'clock, when the first contingent of the Guards left barracks, the streets were full of friends and well-wishers of the soldiers, and at eleven o'clock, when the 3rd battalion of the Grenadiers started, Birdcage Walk was so densely crowded that it was difficult to move. Many of the men, women, and children marched from there to Waterloo Station with the troops. The police, partly from powerlessness, partly from kindness, gave up trying to keep the crowd back, and it was strange to see the women hanging on to the soldiers' arms, the children clinging to their hands, and the civilians carrying the kit. The steps and windows of St. Thomas's Hospital were thronged with nurses, who waved their handkerchiefs, and called out good wishes to the men as they passed.

---

Later in the afternoon came the news of the first victory at Glencoe, which made the services on Sunday seem one long thanksgiving, except when we remembered those who had just laid down their lives, and then our eyes filled with tears. Whether Sir William Symons lives or dies, his name will be always a household word, and when I remembered how he had learnt to fight on South African soil in the sad experience of Isandula—which experience probably did so much towards the present victory—my thoughts reverted to Cowper's hymn:

> "God works in a mysterious way
> His wonders to perform."

Since Saturday the papers have been full of war news. A second victory at Elands-laagte, gained by Sir George White and General French; then a short period of anxiety lest the men at Glencoe, with their sadly reduced numbers, should not be able to hold their own against the Boers; and then the welcome news that General Yule had been able to effect a junction with Sir George White—these formed the important items of intelligence from the front.

---

I have just had a private letter from relatives in Durban which has made me feel more thankful than ever that the British arms have so far been successful in South Africa, even though the victory has been gained by such a disastrous loss of British—and may I say also Boer?—lives. The letter is so interesting that I transcribe a portion for your benefit. My cousin says: "We are all extremely busy. Durban is very full of refugees, and though the Mansion House Fund will help those of the poorer class, there are great numbers of teachers and clerks who have been obliged to leave their work and are homeless and penniless. These are very difficult to reach, because they will not beg for assistance, so we have amongst us formed a Ladies' League and are doing all we can to help them. As many as possible have been taken into our house, and our neighbours have done the same. The mental distress of many of the refugees is almost as sad as their more material troubles. One girl who has just left us has three brothers in the Free State. Two of them have announced their intention of refusing to be commandeered to fight against the English, a refusal which renders them liable to death or imprisonment; the third is a regular Pro-Boer, and will be glad to fight directly he gets a chance, so we have all the elements of a civil war. As to the natives, there is scarcely one who believes the English will win, they have lost all faith in Great Britain being able to protect them from Boer oppression, and are prepared to sit stolidly down and wait till the Boer rules all the land. You who are safely housed in England cannot realise what their want of faith in the power of the English means to us. We have good servants, but their tone to us has changed horribly the last month. England's prestige is *nil*, the natives are everywhere beginning to be insulting, and the Boers are venomous. Fancy one farmer near Harrismith said openly the other day that his only wish was 'to shoot every Englishman, and that if he were wounded and an English nurse offered him brandy, woman or no woman, he would shoot her if he could!' Many of our friends are still in Johannesburg, and we are very anxious, as one, a doctor, is known to have been placed by the Boers upon their list for extermination, and another was warned by the commandant of the Johannesburg forts to send his wife, who is a Dutch lady, away—she has since left—because if the Boers found they were losing they had orders to bombard and destroy the town of Johannesburg!"

### A Book and its Story.

### MR. CROCKETT'S NEW BOOK.

MR. CROCKETT has written nothing better in its particular way than his latest book, "Kit Kennedy."* It has all the charm of that delightful story, amongst the earliest of his writings, "The Lilac Sunbonnet."

The theme of "Kit Kennedy" is woven into scenes dramatic, humorous, and pathetic. Perhaps, on the whole, the tragic incidents exceed the lighter elements in the development of the plot and the evolution of the hero's character, but this is as it should be, if a book is interesting, with a *human* interest which holds the reader and makes him, for the time being, at one with the author.

The story opens in the early dawn of a midsummer day, when "Christopher Kennedy, scholar and gentleman, has aroused himself to go forth to meet with Lilias Armour. It was a strange time for wooing, yet their only opportunity; for Fate, which takes upon itself to interfere with all things, had made Christopher classical master in the academy of Cairn Edward, and Lilias, the daughter of his chiefest enemy, Matthew Armour by name, farmer in the moor farm of Black Dornal, and ruling elder in the Cameronian congregation called the Kirk on the Hill."

That Matthew Armour had his own grave reasons for being Christopher Kennedy's "chiefest enemy" goes without saying; nor was the title of a "blasphemous and ribald man" excessive, when allowance is made for parental acridity in designating one, who was a possible, if not altogether acceptable, suitor for his daughter's hand. "Foolish in all else, Christopher Kennedy was at least wise in love—that is, of the object of love—wisdom be to win other love, and not to hold it worthily when it is won." Lilias, alas! did not in any way, inexperienced and self-willed as she was, gauge the character of her lover; fascinating to a degree, as only those intellectually endowed can be, when in addition to physical attractions is added a total disregard to honour and morality, so deeply hidden under a strong emotionalism, that it is impossible for any but those equally unstable to realise the depths of its unprincipled self-seeking. To Kennedy, "scholar," in fact, but "gentleman" in name only, "Lilias," the Elder's daughter, "the desire of his eyes," had so far given her heart that her father's adverse verdict of his character came too late to influence her love for him. For she "was so holden in the toils of the schoolmaster's bright glances and loving words, that not for father or mother, kirk or Covenant, would she break the bond." In the simple happy home life of Black Dornal Lilias had had been as peaceful as it was possible for her to be, "lifting a light-heart carol level with the larks, and laying her head in as lowly a nest with the falling of the night, that is, till Christopher Kennedy came by, and the song ceased." Then in a moment all was changed. The old life grew inexpressibly dull, not to be thought of, or returned upon for a moment without a shudder—a dreary waste of time, wanting alike in profit, beauty, or happiness. This being the condition of things, it was not surprising that with very little persuasion Kennedy prevailed upon Lilias to marry him secretly. But he deserted her, and she returned to her father's farm, from whence her boy, Kit Kennedy, junior, goes forth later to seek his fortune elsewhere, and so to become the hero of the story. In the meantime, Lilias, finding that her marriage lines are false ones (Kit's father having a wife alive at the time they were made), is persuaded some four years afterwards to become the wife of Walter MacWalter, the owner of Black Dornal Farm. Miserably unhappy, she drags out her days of bondage, her only joy being in covert meetings with her son, "Kit," who lives with his grandparents, Macalister being insanely jealous of the child, and not allowing him to have known access to his mother.

* "Kit Kennedy." By S. R. Crockett. (Publishers: T. Clark and Co., J. Fisher Unwin, London.)

There is a touching glimpse of the mother and son on their way to the school house, where Kit is to be placed under the instruction of Dominie Duncanson. "Kit Kennedy admired his mother above everything on earth—and loved her too, almost as much as his red dog Trusty. So they went out over the heather, only these two together. Lilias stepped sedately and stilly along the rude moorland track, her head a little bent, her eyes but vaguely taking in the purple of the hillsides and the misty blue of the valley lakes. Her face was full of a great, still sweetness, like that of a woman who has indeed won her way to an isle of rest, but through an ocean of pain. And the washing of the waves of sorrow had swept that countenance clear of self." It is after leaving school on this first day on his homeward way that Kit finds a crowd of boys ill-treating a drunken tramp. He "goes" for the principal assailant, who in return attacks him, and Kit inaugurates the occasion by his first fight, in which tenacity and pluck are conspicuous—qualities by which later he was distinguished throughout his hard, uphill struggle in the school of experience. It is with difficulty that the combatants are separated, and then "a hand was laid on Kit's head—an unsteady hand, a hand with long, lithe fingers, a gentleman's hand, spite of signs of recent manual labour. It was the drunken tramp, who had straightened himself and had stood with a certain wayward, swaying dignity, by Kit's side."

Kit's anger melted and his pity came back.

"Can I help ye?" he said. "Tell me where you are going."

"To the last refuge of the unwise," the man answered, smiling wistfully; "the hotel of the misfortunate; the sanatorium of those who have lived not wisely, but too well. Set me on my way to the poorhouse and I will bless you, my boy; but first I will shake off the mud of this ungrateful village from my feet." . . . . At this moment Kit's mother was seen coming down the white road towards them. The tramp gazed a moment at her, standing as if petrified, and then instantly a wondrous change passed over his countenance. Kit leaves him, called away by his mother. The tramp moves on. Tremblingly he took from the inner pocket of his coat a faded pocket-book and unfolding a packet containing some faded hare-bells, on the cover of which was written, "Given me by Lilias." "Bless God she did not know me to-day." . . . . "He lifted the paper half way as if to kiss it, and then he restored the whole with reverent care to his pocket." Throughout the story to its close there are vanishing glimpses of Kit's father, who finds work on a farm in his son's vicinity, where he has himself found employment away from home, and under his instruction he becomes sufficiently proficient to win a Bursary at Edinburgh University. Of his student life and his meeting with Mary Bissett, a striking little person, of their love episodes and the kind Fate which ultimately steps in and makes everyone happy, we have graphic and touching descriptions. Their full charm can only be realised by reading the story of "Kit Kennedy" for ourselves.

### OUR HOSPITAL CONVALESCENT FUND.

THOSE who have helped us by contributions to THE HOSPITAL Convalescent Fund will be pleased to hear that we have received a grateful letter of thanks from Nurse Bessie for a grant. She had had a long and expensive recovery from a serious illness, and had exhausted her savings when she asked help. The doctor's certificate showed that she needed further rest before she was fit for work. The pleasure of helping such a case is enhanced by the fact that she is one of the nurses who so splendidly fought the typhoid epidemic at Maidstone.

### Steam Disinfection Apparatus.

SOME CONTINENTAL METHODS.

The apparatus of Henneberg, of Berlin, is perhaps the typical form of that class of disinfector in which the current steam used is produced in a separate boiler and is admitted from above downwards. Hot air is first admitted from above to heat the articles to be disinfected, and then steam, the latter being directed upwards by the guard over the steam inlet, and both the hot air and the steam find their outlet below. The chamber has no double jacket, but is framed in non-conducting material so as to prevent unnecessary loss of heat.

Another steam disinfecting apparatus we shall mention is the Geneste-Herscher stove, one that is, perhaps, more largely used on the Continent, especially in France, than any other. It is made in two forms, horizontal and vertical, so that it can be adapted to a variety of purposes. The horizontal is the larger apparatus, and is suitable for disinfecting on a large scale, whilst the vertical can be used for the double purpose of disinfecting small quantities of clothing at a time or for sterilising surgical instruments and dressings, and hence is invaluable to small hospitals. The advantages claimed for this apparatus are that a temperature of 239 deg. Fahr. can be easily obtained with steam at 10 lb. pressure, that it disinfects reliably the most resistant organisms in fifteen minutes, and that it fulfils every condition of perfect disinfection. It is very economical, using less than 2 cwt. of coal in ten or twelve hours' working, and this quantity can, if desired, be still further reduced, as steam from any other existing boiler may be used; and it is declared to be extremely favourable to the drying of all objects. The disinfecting chamber is a wrought-iron cylinder without jacket or other means of superheating the steam, but it is covered with wood outside and lined with some non-conducting composition inside, and thus the cooling of the steam through the walls of the chamber is reduced as far as possible. A row of steam tubes runs longitudinally inside the stove at the bottom, and in the larger size apparatus at the top also, to warm the chamber before and during the process of disinfection, and also to heat the air which is subsequently used to dry the thicker articles. The apparatus is fitted with gauge and safety valve, so that when the required pressure has been attained, as indicated by the gauge, steam at once escapes through the safety valve and attracts the attention of the attendant. When the chamber has been thoroughly warmed the articles to be disinfected are run in on a wheeled carriage along rails within the stove, and the door is secured. The steam enters by a "sparge pipe" which is placed longitudinally inside the stove near the top, and is fitted throughout its length with a screen to secure the projection of the steam to all parts of the chamber. As soon as the steam meets the articles to be disinfected it condenses, and the decrease in volume thus produced sucks more steam into the pores, which in its turn condenses, and this goes on until there is no difference between the temperature of the steam and that of any of the contents of the stove. In consequence a film of water is formed throughout the articles under a pressure just sufficient to prevent its evaporation, and advantage is taken of this fact to get rid of the air originally in the pores of the articles by shutting off steam from time to time by means of a valve. The sudden reduction of pressure thus caused re-evaporates suddenly the condensed steam in the articles, and what was water in the pores becomes steam and sweeps before it the contained air. The process of disinfection is completed in about fifteen minutes in the case of articles of ordinary thickness, which are then found to be perfectly dry, whilst such things as mattresses are replaced in the stove, which is again closed and hot air is admitted, and in about five minutes they are quite dry.

The vertical apparatus for small quantities of clothing, &c., consists in an open water vessel inside a closed cylinder, having a cock on the top and also a safety valve, and inside are fitted wire cages of various sizes to hold the articles to be disinfected, and steam is produced by means of gas or a spirit lamp. The steam is first allowed to blow off through the cock for a short time to drive out all air; the cock is then closed and the steam enters the apparatus, and when the required pressure has been attained escapes through the safety valve. If thick articles are to be disinfected the gas or lamp can be turned down from time to time and the cock turned to blow off steam till the gauge falls to zero, and thus extract all the air from the articles. A temperature of 120 deg. C. can be reached; but this stove does not dry the articles afterwards, and hence its construction can be much simplified. The stove can, however, be used also as a hot-air chamber, when the temperature must be raised to at least 150 deg. C., and exposure of articles must extend over an hour, so as to provide for the irregular distribution of heat.

Van Overbeck de Meyer's disinfector is one of the simpler forms of apparatus and is designed for use with steam at atmospheric pressure. It is said to work well and has the additional advantage of being cheaper and less complicated in construction than some others. The chamber is provided with a jacket, and steam is generated first in the jacket-boiler and passes thence into the interior of the chamber through an opening in the top, and thus is provided what is so essential to thorough disinfection, viz., a current of steam to displace the air that is already existing in the articles to be disinfected when they are first placed in the chamber. As the steam is not under pressure the temperature does not exceed 212 deg. Fahr., but judging by the careful experiments that have been made penetration appears to be little less rapid than it is at a higher temperature and pressure. Drying of the disinfected articles afterwards is provided for by means of a current of hot air, which can be passed through the chamber after the steam has been shut off, but it is rarely found necessary. The combination of boiler and disinfecting chamber in the apparatus gives greater economy of heat, more complete absence of condensation and greater economy of space. This combination is effected in a double-jacketed chamber by filling the lower half of the space between the two jackets with water, which thus acts as a boiler with a furnace below. Also the use of current steam has demonstrated the advantage of steam being admitted from above downwards in displacing the cooler air contained in bulky articles, in reducing the amount of condensation and wetting, and in increasiag the penetration of the steam.

Dr. H. Rohrbeck's disinfecting apparatus is constructed on a somewhat different principle and seems to be an excellent stove. Steam is made to pass over a cooling surface before it is allowed to enter the disinfecting chamber, and by this means it becomes thoroughly saturated first, and afterwards the steam in the disinfecting chamber is condensed, and consequently it imparts its latent heat to the objects, thereby producing a vacuum, and thus the air is sucked out of their interior and the efficiency of the disinfecting process greatly increased. When steam is again allowed to enter it readily forces its way into the interior of the objects and completes their disinfection. After the conclusion of the process the disinfecting chamber acts as a drying oven.

### The Nurses' Bookshelf.

[We invite Correspondence, Criticism, Enquiries, and Notes on Books likely to interest Women and Nurses. Address, Editor, THE HOSPITAL, (Nurses' Book World), 28 & 29, Southampton Street, Strand, London, W.C.]

HINTS TO PROBATIONER MIDWIVES. By ANNIE S. PRUETT, Superintendent of Nurses, Union Infirmary, Sunderland. (Sunderland: Thos. Todd. 1899. 19 pp.)

THIS little book forcibly recalls the fact that, however intelligently a nurse may perform the practical duties of the lying-in room, she does not thereby acquire the faculty of setting them forth clearly upon paper. There are many points, doubtless familiar to the author, about which fuller information is necessary before the book can be recommended as a safe guide to one entirely ignorant of midwifery. Thus, the strengths of the antiseptic solutions employed and the indications for medical assistance are vaguely mentioned. The blank pages provided for notes will doubtless be useful in supplying these and other omissions.

A HANDBOOK WITH HINTS—PLAIN, HOMELY, AND PRACTICAL—FOR THE NURSERY. By J. MACLEAN CARVELL, M.R.C.S. (London: George Barber. 1899. 70 pp. Price 1s.)

INTO this little treatise on nursery hygiene the author has contrived to crowd much miscellaneous information—some of it excellent, so far as it goes—on the management of children. There are also brief statements of the laws as to vaccination and registration of births which, if somewhat unusual in such a volume, may occasionally prove useful. What is chiefly to be regretted is the want of arrangement of the various subjects, and the strange disparity of space allotted to them:—a brief six lines on the eyesight of school children comes between registration and the use of the tooth-brush; the treatment of burns after nursery furniture.

The weights of infants at monthly intervals for the first year, and of children according to their height, should prove of undoubted value, as a steady gain in weight furnishes perhaps the best test of a child's condition, and any arrest of this should be regarded as a matter for investigation.

In the appendix some useful hints on the care of children in tropical climates are given, with a suggestive letter from a friend of the author on the study of the idiosyncrasies of children, even belonging to the same family, and the need for careful individual attention, both moral and physical.

In order to facilitate this study of children, especially when ill, the author has adopted the novel expedient of printing the text on one side of the page only, leaving the other for annotations, while further blank pages are provided at the end for notes by the mother or nurse and the instruction of the medical attendant. The whole forms a neat volume which may well find a place on the nursery bookshelf, and its extremely moderate price places it within the reach of all.

FROM A NURSE'S NOTEBOOK. By HONNOR MORTEN, Author of "The Nurses' Dictionary," &c. (London: The Scientific Press.)

MISS MORTEN'S sketches of nursing life are worth reading for one good reason, namely, that she knows what she is talking about, which most story-tellers who attempt the like do not. The author warns us that the first of the "Notes" was written in 1884, and that what was true of the hospital world of fifteen years ago is not altogether true to-day. We might add that the attitude of the author to life is not the same to-day as it was when she first began to jot down her impressions of nursing work. The earlier fragments of diary with which the volume opens show the same observing eye and sympathetic mind as the chapters that follow, but there is a touch of the pose, the self-consciousness of youth. In those days "Drusilla" noted and criticised her own emotions, as, after her training was complete, she criticised those of others. 'Tis the common fault of diarists, especially when they are young women, and, indeed, diary-keeping may almost be set down as as a symptom of neurosis. We like Miss Morten best when she is speaking of others. The story of "Patience Angelica," the child whose innate violence of temper, developed in an atmosphere where it was a matter of pride to be "took up before the magistrate," led her to a reformatory, and to suicide there as the only way of gaining the freedom she craved, is perhaps the tale that appeals to one most. In "A London June" one cannot but think that Merston is rather a poor creature, dying for love of a woman who regards him merely as a convenient "chucker-out" of servants she does not like. The sketch of insomnia will probably be less fresh to readers than the rest of the volume, and the "Nineteenth Century Neurotic," whose character is probably compounded of several originals, is pretty much like the neurotics of previous ages. But in "Where's Sister?" Miss Morten strikes a note altogether new, serious, and noble. The sister of a children's ward, loving her little patients and her work, gives it up to go and nurse in a lock hospital. Perhaps Miss Morten's lay readers who have never been in such a place will hardly understand how great this a sacrifice must be, though they will instinctively share the shrinking with which all the visitors to the hospital heard of Sister Rachel's choice. Well does the author cry out, "Hysterical women who tell the public they are going to devote their lives to the nursing of lepers are praised and fêted and made famous. My God! those sentimental fools are to be held up as heroines, while here at our gates is the quiet self-sacrifice, the daily danger of those nurses who face contagion in comparison with which leprosy is almost clean!" The last chapter of the book contains some admirable notes of slum life. Take this: "There is a little chemist's shop at the corner, and all Saturday evening a stream of children go in and ask for 'a 'aporth o' 'air-oil and a pennorth o' insect powder.'" But why did Miss Morten fill up interstices with scraps of Schopenhaueresque philosophy, which is not new, and is not true, and does not matter? For example: "The gnat is destroyed by the spider, the spider by the bird, the bird by the man. And so man imagined something bigger than himself, for he saw that he also was subject to destruction, and he fashioned God in his own image." This is smart, but it hardly solves the whole mystery of Deity. And what does Miss Morten mean by the outburst with which she concludes her book? "Harken, O Eve, mother of us all, greatest and grandest of women; you who have been maligned all down the ages, know at least that one of your daughters blesses you, and proclaims that your choice was good. To you, Oh Eve, we owe it that we are as gods, and not as children playing in the garden—that we know the good and the evil, and are not lapt in ignorance and lust. Man had stayed for ever in uninquiring peace, but to you was given strength to grasp the apple, to proclaim that woman at least prefers wisdom and the wilderness to idle lasciviousness in Eden." A protest like this raises questions in one's mind. Would man have stayed in uninquiring peace? Certainly, since he ate his half of the apple he has not lagged behind woman in the study of evil. Is lust as well as ignorance a characteristic of the women who do not go forth into the world to battle like the Amazon? Is the domestic woman either idle or lascivious? Miss Morten seems to know Hoxton. Let her ask the wives and mothers of Hoxton—or any other place.

### Zenana Bible and Medical Mission.

The farewell meeting, to take leave of three new missionaries, and of twelve ladies returning to their work in India, was held at Morley Hall last week. The Rev. H. E. Fox occupied the chair, and gave an earnest address. The missionaries were introduced by the Rev. A. R. Cavalier (secretary), and some of them were called upon for a few words, amongst them being Miss Gray, L.R.C.P., S.Edin., who is returning to her work at Victoria Hospital, Benares. She said that the natives in India did not look upon sick nursing as being a proper, right, and fit thing for ladies of any position to undertake, but had it delegated to the poorest of the low caste women, with whom every other woman would dislike to come into contact. In an Indian hospital there was a great deal that was repulsive, and the patients were very tiresome. Our ideas of order and exactitude were so much higher than they had ever been accustomed to; but looking back upon the work done by the nurses, she felt that for some of the best we were indebted to the native Christians in our hospitals. The need for medical women was great, she added, and Lord Roberts had stated that two thousand medical women were wanted in India. There were not two thousand medical women altogether—they had only just reached the hundreds. The preaching of Christ in the hospitals did not lessen the attendance, and she felt strongly the great value of the careful and systematic Evangelistic work in the hospital. After a few words from Lord Overtoun, the Bishop of Honduras delivered a valedictory address.

### Concerning Teeth.

By a Correspondent.

Of all the ills that flesh is heir to, I am sure most nurses will agree that toothache and neuralgia, often another name for the same thing, play no small part. The care of the teeth is important, nay, essential to those who have plenty of strain on their nerves in other directions, and no one can be said to be physically fit for continuous and hard work whose teeth are in an unsatisfactory condition. Our teeth are not as good as those of our forefathers, or rather, they do not last as long. Why is this? Perhaps, as some say, the strain of modern life, more artificial food, alum in bread, &c., may have something to do with it; but I am inclined to think the abolition of the toothpick has a good deal more. This useful little instrument used to play an important part in polite society; now it is nowhere. But there is no reason why teeth should suffer because fashion has changed.

All nurses know that there is nothing so conducive to decay and all its attendant unpleasantness as organic matter that has been neglected. The tiny particles of food that lodge between the teeth make an excellent place for the germs of decay to flourish; the brush does not dislodge them all, because, as a rule, the teeth are too close together, and these are the first to "go," so we must not pin our faith to the tooth-brush, even if properly used. If the sides and the back of the teeth are clean as well as the front, especially at night, when, the mouth being shut, there is not free access of air, then we may be sure that the germs have at any rate no encouragement. I have found the most practical way is to take a fine piece of silk or cotton. Sometimes a thread unravelled into three is as much as will pass between the teeth. Draw this thoroughly up and down, and backwards and forwards, using a fresh bit when necessary. If you have not done so before you will be surprised to find how many particles have escaped the brush. "But what a nuisance to do all this every night," I fancy some tired nurse exclaims. Well, dentists' bills are a nuisance, and so is toothache. I should prefer to spend ten minutes daily in warding off both.

### Everybody's Opinion.

**[Correspondence on all subjects is invited, but we cannot in any way be responsible for the opinions expressed by our correspondents. No communication can be entertained if the name and address of the correspondent is not given, as a guarantee of good faith but not necessarily for publication, or unless one side of the paper only is written on.]**

### "HOURS OF REST FOR PRIVATE NURSES."

"R. A. G." writes: When nursing single-handed, I usually have supper at 9 p.m., come on duty 9.30 p.m., and leave duty at 11 a.m. Then I go out until 12 noon. I have my dinner when I return and go to bed by 12.45 p.m. I rise at 8.15 p.m. and go to supper. I find people would rather give me an early dinner than be kept up late at night, although some object, and then you have to get it when you can and lose your rest, for very few persons like being in the sick room so late as 11 p.m.

### BABY'S CORD.

We have received a large number of letters on this subject, of which we can only find room for a selection, not necessarily always the best, but the most varied in interest. But we have to thank "Nunclynes," "Successful District Nurse," "One Who Has Tried It," "Nurse C. B. C.," "L. O. S.," "Welsh District Nurse," "A City of London Nurse," "E. J.," "Nurse Mab," "Leslie," "Nurse Elizabeth," "M. S. P.," and many others, for their contributions.—[Ed. *T. H.*]

"Yam" writes: In answer to "Perplexed," personally, I think the idea of not washing and dressing the cord every day most repugnant, and most certainly not aseptic. We were taught to wash and powder the cord thoroughly. It was then folded in a little piece of clean old linen about three inches square, and slit half-way down the middle, which made it fit round the cord nicely, and so prevented it irritating the infant when it became hard and dry. The daily bath may retard the quick dropping of the cord, but if it is carefully dried and powdered with equal parts of starch and boracic it does not do so to any serious extent.

"C. E. W." writes: In answer to "Perplexed," I am sure she would find it a better way to dress the cord every day, as it is apt to get wet with urine, or even perspiration. We were taught to put the baby in the bath each day; temperature of the water 98 deg. (Herman). This would of course moisten the dressing, so that it could easily be removed. Diseases of the navel are in almost every case preventable. If the binder is not put on firmly the first time the cord is apt to hang down, and if it is at all heavy it is sufficient to cause the navel to protrude. If from any cause the cord is allowed to remain damp it will become ulcerated. The navel should be well washed and dried every day (if at all offensive a little Condy should be used), afterwards well powder (not simply dust) with a mixture of starch, zinc, and iodoform.

"Anxious" writes: I am much interested in the letter from "Perplexed," having experienced the same trouble with the cord in many of my private cases, and it was not until I had the privilege of nursing in a doctor's family that I succeeded in having a satisfactory navel cord with my young infants. This is my plan: I take a piece of old linen about four inches square, make one cut from middle of side to the centre, which should be slightly notched. Rub a little boracic powder round the umbilicus and about an inch beyond, then gently lift the cord, place the linen around it, fold the two cut edges over each other, coil the cord round upon the linen, the cut end being upwards; sprinkle more boracic powder, turn the two folded ends of linen over coil, bring the lower flap of same up, fold firmly down, doing likewise with side pieces, thus making a pad. Cut three graduated pads of absorbent wool about one and a half inches square, place them on the pad, and affix the binder in the usual way, taking great care not to drag the dressing. Dress the cord in this manner every morning, and wash it with warm boracic lotion before applying a fresh pad. The cord thus treated should drop off not later than the fourth day; then make a pad of absorbent wool, covered with soft rag, and keep this changed until the tenth day, when all chance of protruding umbilicus is over. Do not be tempted to place the child in a bath until the sixth or seventh day. I may add that the cord is often dragged out of place through the mother, grandmother, and affectionate aunts lifting the child improperly; they should be instructed to place their hands under the body, and then lift. When practicable, it is safer not to allow anyone to touch the child until the cord is off.

"FOR THE BABY'S SAKE" writes: In answer to "Perplexed" I have always found the following the best way to dress the cord: Cut a square of lint with a hole in the centre and a slit through one side, place this round the cord with the cut side uppermost, slightly overlapping the cut edges. Then put plenty of powder on the lint (I always use equal parts of boracic acid, starch, and zinc), lay the cord up in the usual way, fold the lint over from side to side, and turn up from the bottom. When this is done raise the dressing slightly and apply the powder round the insertion under the lint. I consider it most necessary to change the dressing every day, and by using plenty of powder it is easily removed without any moistening or pulling on the cord, and for this reason I have found cutting the slit in the lint a great advantage, as you can unfold and remove gently from either side. There is no reason why the baby should not go into its bath daily from the first; I always wash it all over on my knees, then remove the dressing from the cord, put it in the bath, and carefully bathe round the cord to remove any remaining powder, after which dry thoroughly, and dress in the same way each day. I can confidently say that with this management I have found the cord come off from the fourth to the eighth day, leaving the navel in a most satisfactory condition. The flatness of the navel depends very much upon the evenness of pressure produced by the binder, which if put on slanting at first, that is, from the child's left shoulder down to the right hip, then brought once straight round the body, and the end slanting in the same way, will give firm even pressure where needed. A perfectly fitting binder, if properly put on does not slip out of place. I consider a deep binder much better than a narrow one.

"E. A. B.," who encloses a piece of linen cut in the requisite shape, writes: In answer to "Perplexed," may I give my method? I am a midwife of many years' experience, and was trained at Queen Charlotte's Hospital, with L.O.S. diploma. At Queen Charlotte's I was taught to use Fuller's earth for dressing the cord, and a piece of fine linen (an old handkerchief does) cut in the shape I enclose. I was also taught to bathe baby all over, putting it into the bath every day, changing the linen each day as a matter of course. I have always carried out this teaching, having never found a better method. I have never had a bad navel, though, rarely, through constitutional weakness, there may be a little redness. If the cord seems moist, as it will sometimes, and smells offensive, I substitute starch and boracic powder for Fuller's earth, bathe the navel and cord with a little weak Condy, after taking the child out of the bath, and put a piece of boracic lint over the other dressing before applying the binder. The daily bath does not soften the cord, and the soiled linen soaks off in the bath. Cotton wool is a very bad thing to dress the cord with. Firstly, the hairs or fluff will adhere to the navel, and secondly, cotton wool if it gets wet dries hard and stringy. Some cords drop off sooner than others. I have known them come off on the third day, and I have known them to remain until the tenth day. One cannot always get a flat navel, as sometimes the skin grows as much as half an inch up the cord. In this case the navel is bound to be large, and requires a good pad on for several weeks. There is usually a little moisture from the navel for a week after the cord falls off, but if it is well powdered, and the linen pad made so large that the flannel binder does not chafe the tender skin, this quickly disappears. Linen should always be applied next the skin till the child is a month old. From my experience the daily bathing and changing of the linen and the thorough powdering accelerates, not retards, the dropping off of the cord. Should there be anything in this that "Perplexed" does not quite understand, if she writes to me, care of the Editor of the "Nursing Mirror," I shall be pleased to help her, and would forward her, if she desired, the piece of linen cut into the right shape.

### "ONE OF THE FAMILY."

"VERA" writes: I cannot help thinking that it is the nurses who go into a house with the fixed idea that they have a right to be treated as members of the family who make unnecessary trouble for themselves when they are not so treated. "A Nurse from the West" seems to think me a little hard, and I quite think with her that people ought to try and make a nurse's stay in their house as pleasant for her as possible, but I still believe a nurse herself would be happier if she did not go to a case expecting too much from the family. As "M. S." says, it is a time generally of great anxiety and trouble for the household, and after all a nurse has not come there for her own comfort or pleasure—as a guest to be entertained—but as a helper, a comforter, and to be "an angel in the house." If the nurse is going to begin by standing on her dignity and disliking to do things which she is pleased to call "unprofessional," such as "cooking the meals and helping to keep the house in order," she had far better try to find for herself another vocation. Nursing is obviously not her line. I speak from the standpoints of nurse, patient, and patient's friend, and from the vantage ground of each of these positions I say, frankly, I do not believe in a nurse talking grandly of the dignity of her profession as making her unable to be generally useful. The nurse who is most helpful, most ready to turn her hand to anything, most anxious to save trouble and do all she can to spare extra worry in an already worried house, is the nurse most likely to be received as "one of the family." Still I confess it puzzles me a little to imagine why nurses covet this position so greatly. I think after all there is a higher thought for the nurse than this. There is a motto I would recommend everyone who nurses to make her own, because then she will forget her petty dignity or any small slights she may receive. The motto is, "I am among you as one that serveth," and the nurse who carries those words always with her will only long to serve, like Him who though "the greatest man" was also "the servant of all."

### IS THE NURSING PROFESSION OVERCROWDED?

"LADY SUPERINTENDENT," St. Leonard's-on-Sea, writes: Having seen in your columns an answer to this question, I, too (having an intimate knowledge of the nursing world of Paris), shall be greatly obliged if you will allow me space in your paper to make a few more observations on this subject. For instance, a statement is made that there is a good opening for trained nurses in Paris and Berlin, and thus employment could be found for the English nurse, with whom London is already too well supplied. With regard to nursing in Berlin I can give no opinion, but Paris and many of the English nurses I know, and I can only imagine that the nurse who made this statement did so without knowing anything of the nursing centres already established there. She suggests the starting of a home for English nurses in Paris, and speaks as if the English nurse were an unknown quantity with the French doctor; but this is not so. In the Rue d'Aquelleau (Faubourg S. Honore) is a flat hired by six certificated nurses who were trained in some of our best English hospitals. This little settlement is very well known and the nurses are much appreciated by the French doctors. There is another nurses' settlement in the Rue d'Amsterdam. There are nurses who are working independently for themselves, and there is more than one institution for trained nurses. There are, of course, times in Paris, as in London, when the demand for nurses is great, and the supply for a week or two seems insufficient; but also, as in London, there are times when nurses have to wait weeks for a case, and, what is worse, there comes to Paris (and never to the same extent in London) a time when there is no one left in Paris—when English and Americans alike have fled to cooler places. This condition of things lasts at least three months each year, and during all this period there is no work for the English nurse. Before I close I would warn any nurse attracted by erroneous statements to Paris not to go there until she is sure of work; has friends to whom she can go, or an introduction to some well-known doctor who will give her work. Rents are high, food is not cheap, and more than one nurse (I speak as I have heard of them) has used up all her savings while waiting to find a footing with a French doctor who has already a staff of trusted English nurses on his list.

### For Reading to the Sick.

"Come unto Me all ye that labour and are heavy laden, and I will give you rest."—*St. Matt.* xi. 28.

"When He giveth quietness who can make trouble?"—*Job* xxiv. 29.

"Come unto Me
And I will give you rest." "Once more the voice
Is in my ear. It seems to echo now
The mournful hope that death should give me rest;
And yet I know this is no dream-like sound
Of sad Death making answer. This the voice
Of Life and not of Death!" . . . He spake
Of giving rest, and on the bitter cross
He gave the promised rest! O Christ, the King,
We also wander on the desert-hills,
Though haunted by Thy call, returning sweet
At morn and eve; we will not come to Thee
Till Thou hast nailed us to some bitter cross,
And made us look on Thine; and driven at last
To call on Thee with trembling and with tears—
Thou lookest down in love, upbraiding not,
And promising the kingdom! —*B. M.*

**Reading.**

The mind may be restless, while the body is still; too well do I know that. In my body I cannot but be still, for I am not to stir from my bed; but my thoughts are often disturbed, and I find it difficult to lie quiet.

These words seem to speak to me from God, "Be still." It is not His will that I should be tossed hither and thither in my thoughts. It would be much for my comfort in all ways to keep a quiet mind; but it is my duty too, for God says to me, "Be still"; be still in mind as well as in body.

"Be still, and know that I am God." My own restless mind troubles me with the thought that things cannot go on well without me. All the various things that I was busied about, the affairs I used to manage, the concerns in which I was chief, how can they fail to be neglected and fall to ruin, now that I am laid aside? "Be still, and know that I am God," is the answer; if those things were right and useful things, cannot I do them without thy help? If those affairs and concerns were in truth ordered by Me, and thou wast but My instrument, cannot I find some other instrument? Must all come to a standstill because thou art laid aside? Thus God seems to speak to me in the words, "I am God." He seems to say: I have countless instruments; all that is needful for My service, nay, all that is for thy good, I can do; true, I have used thee hitherto in My service, but thou art not necessary to Me; I can do without thee; therefore, "Be still."

Give me humble resignation; work in me by Thy Spirit a perfect willingness to lie still as long as it is Thy will that I should, and lead me to rest tranquilly in Thee.—*Bourdillon.*

When the rest of faith is ended, and the rest in hope is past,
The rest of love remaineth—Sabbath of life at last.
No more fleeting hours, hurrying down the day,
But golden stillness of glory, never to pass away!

Time, with its pressure of moments, mocking us as they fell
With relentless beat of a footstep, hour by hour the knell
Of a hope or an aspiration, then shall have passed away,
Leaving a grand calm leisure—leisure of endless day!
—*F. R. Havergal.*

### To Nurses.

In order to increase and vary the interest in the *Mirror*, we invite contributions from any of our readers in the form of either an article, a paragraph, or information, and will pay a minimum of 5s. for each contribution. All rejected manuscripts are returned in due course, and all payments for manuscripts used are made at the beginning of each quarter, *i.e.*, January 1st, April 1st, July 1st, and October 1st.

### Notes and Queries.

**The contents of the Editor's Letter-box have now reached such unwieldy proportions that it has become necessary to establish a hard and fast rule regarding Answers to Correspondents. In future, all questions requiring replies will continue to be answered in this column without any fee. If an answer is required by letter, a fee of half-a-crown must be enclosed with the note containing the enquiry. We are always pleased to help our numerous correspondents to the fullest extent, and we can trust them to sympathise in the overwhelming amount of writing which makes the new rules a necessity.**

**Every communication must be accompanied by the writer's name and address, otherwise it will receive no attention.**

*Help.*

(39) I write on behalf of Sister Eleanor to ask if there is a fund to assist lady nurses who are incapacitated by illness. The case I refer to has failed through overwork, &c. It is feared that cataracts are forming, and it may be two years before she can have an operation. This necessitates more expense than she can meet. Do you think she could possibly be helped with the sum of £10?

If Sister Eleanor does not belong to the Royal National Pension Fund we fear she must fall back on private help. She can do no harm, however, by applying to the secretary, the Junius Morgan Benevolent Fund, 28, Finsbury Pavement, E.C.

*Plague Nurses.*

(40) Will you kindly tell me who sends the plague nurses to India?—*F. L.*

The Under-Secretary of State for India, the India Office, S.W.

(41) Kindly tell a nurse when the next detachment of nurses for plague duty in India will be sent out.—*German Jerry.*

We have not heard yet that any date is fixed for the next detachment of plague nurses to be sent out. See reply to F. L.

*Chest.*

(42) Would you kindly let me know if there is any "convalescent home" in Bournemouth, or anywhere round the Isle of Wight, where I could be received (chest trouble) for six months. Terms must be very moderate indeed. Any particulars you can give me will be thankfully received.—*Nurse Mary.*

If you can afford it, Erith House Institution for Invalid Ladies of Limited Means, Torquay, seems to meet your requirements. Write and ascertain. The National Hospital for Consumption and Diseases of the Chest, Ventnor, Isle of Wight, is suitable as far as we can judge from your letter. There is also the National Sanatorium for Open-air Treatment at Bournemouth. For others, consult "Burdett's Hospitals and Charities" (Scientific Press, W., price 5s.).

*Surgical Aid.*

(43) I am interested in a woman who has been obliged to have her leg taken off below the knee. It would be a great boon to her if she could be assisted to get a cork leg, so that she can follow her profession of cook. I understand there are societies to help such cases. Would you kindly give me any information?—*Alice Thomas.*

The Surgical Aid Society, Salisbury Square, Fleet Street, E.C., would probably help her.

*Teeth.*

(44) A. K. is anxious to train as a nurse, but as her teeth are rather bad she asks us if this will prove a hindrance to her being accepted as a probationer in a hospital.

Not if she goes to a good dentist and has them thoroughly put in order before making her application.

*Deafness.*

(45) A fortnight since someone asked the name of a particular fan used in cases of deafness. I know a gentleman who for several years has found of great service to him a board called the "Audephone." As originally invented it consisted of a sheet of ebonite about 12 in. by 9 in., to be held between the teeth by one edge and bent forward with the hand so as to form a curve, the round part being turned towards the source of sound. A sheet of dry millboard will answer the purpose, and a piece of birch veneer answers better still.—*C. E. H.*

*Home for Children.*

(46) Would you kindly tell me the probable cost of a small home for children; those who are quite homeless, without friends or relations, or from the workhouse would be taken, about twelve in number? Weakly children would have the first offer. What part of England would be the cheapest place for such a home? I am asking for a lady (my present patient) who would like, if possible, to open it in her lifetime, and endow it at her death.—*Nurse.*

The cost of building would depend chiefly on the architect, and that of the up-keep on the scale on which it was managed. "Burdett's Hospitals and Charities" gives the expenditure of a large number of institutions of various kinds with the number of persons benefited, and by a comparison of these it might be possible to obtain an idea of the cost of maintenance per head. It would probably be desirable to take the cost at something between that of an orphanage and of a cottage hospital.

*Stains.*

(47) Can you tell me what is the best method for removing stains (ink, acids, &c.) from polished ward tables and floors?—*Matron.*

Bleach with oxalic acid, then restain and polish.

*Surgical Instrument.*

(48) Can you tell me if there is any good instrument made for a child who has talipes varus to wear? She wore irons, but it was thought best to leave them off. It seems if she had something like Dr. Davis's patent for flat foot, only turned the other way, it would do. There are corns on the outside of the foot through "treading over." The other leg is affected by infantile paralysis, and is a little shorter.—*Era, Weybridge.*

You should consult a specialist about the case.

### Travel Notes.

By Our Travelling Correspondent.

XXXIX.—BRUSSELS—*Continued.*

You will find the trains exceedingly cheap and convenient. The lines extend all round the city and even as far as Laaken, where there is a cemetery fondly supposed to resemble Pére la Chaise. The Bois de la Cambre is a prettier journey, it is in reality a portion of the ancient forest of Soignies. Theatres are good and cheap, pit only 2 frs. Concerts, too, are excellent and very reasonable compared to England, from 1 to 3 frs.

Shopping in Brussels.

Leave your purse at home if you desire to be economical, for the shops are fascinating; the most fashionable are to be found in the arcades such as the Gallerie St. Hubert and in the delightful Montagne de la Cour, an extremely steep street lying between the Grande Place and the Place Royale. There is not a prettier of its kind in Europe. Here you may buy anything in the way of costumes, millinery, jewellery, or bric-a-brac; here the rank and fashion of Brussels disports itself between the hours of three and five, and here carriages, cabs, and even omnibuses go ceaselessly up and down to the accompaniment of most scientific whip-cracking. The shops in the different galleries are not quite so expensive. Visit the Gallerie St. Hubert, close to the Grande Place; from this you can branch off to the others, of which it forms the nucleus.

The Picture Galleries.

Reserve these for a wet day. The older pictures are at the Palais des Beaux Arts, at the corner of the Rue de la Regence, and the more modern in the Place de Musée. There is a small and valuable private collection belonging to the Duke d'Arenberg, but the entrance fee is 2 frs. The Wiertz Gallery stands alone in the world for uncanny weirdness and horror. On no account let anyone go there whose health and nerves are not in good order. It is in the Rue Vautier. Wiertz was a native of Dinant, and undoubtedly had a twist, and a very unpleasant one, in his brain. I think the most striking in the collection is "Napoleon in Hell"; there is indeed grandeur in that, but one can only turn with loathing from such disgusting representations as "Buried Alive" and "Hunger, Madness, and Crime."

The Site of the Famous Ball.

On this subject there is still and always will be considerable controversy, though it would seem to be simple to obtain the information from a descendant of that Duchess of Richmond who gave the celebrated ball on the eve of Waterloo. Some people maintain that it was in the Rue de Blanchisserie, now given up to base purposes, in the old town; Mr. Baedeker announces with an enviable finality that it was in the Rue Royale, and that the house still stands. In any case, we can well fancy the officers stealing off one by one in the direction of the Forest of Soignies, through the silent streets of the old city. What a night of horror for those gracious women and brave men, in many cases parting never to meet again.

The Field of Waterloo.

The best way and the least fatiguing is to visit the battlefield by the coach, return fare 7 frs. It makes the journey at nine a.m. every day but Sunday. A much cheaper way is to take the train to Braine l'Alleud, ticket 75 centimes each way. From the station walk to the Hill of the Lion, a mile and a-half distant. Beggars and touts are insufferable, and if you wish to avoid their persistent company you can take an omnibus there and back, 1 fr. 50. Your interest, like mine, will probably be greatly centred in the château of Hougoumont, now a farmhouse, round whose walls the battle raged with varying success the entire day. Had this important point once been secured to the French the result of the battle would probably have been very different. Once it caught fire, but the brave defenders fought the flames as well as the enemy, and at the close of the day had not yielded one inch. La Haie Sainte, equally bravely defended by Major Von Baring and the Germans, was evacuated after some eight hours' stern resistance because of the failure of ammunition. These two farmhouses, distant from each about one mile, are the most interesting spots on that historic ground. The extensive excavations which have been made to construct the ridiculous mound, with its lion on the top, has so altered the character of the ground that it is almost impossible to identify the various places occupied by the contending armies. Slightly to the south-east is a low-pitched, poor-looking dwelling called La Belle Alliance, near which Napoleon remained the greater part of the day. A mile still further south is Planchenoit, the scene of prodigies of valour on the part of our allies, the Prussians, and where a large obelisk is raised to the memory of the officers and men who fought and died there. The little church at Waterloo contains numerous monuments to the memory of English officers, but the greater number were buried where they fell, and when one considers that the losses of the allies are computed at 14,000, and those of the French at something like 30,000, one is not surprised that even now occasional relics are ploughed up. But the stock is far below the demand, so that the greater part are make-believes. The battle seems still, to our imagination, so recent, that it is hard to believe that no veterans can now survive who were present at that glorious and sanguinary struggle; even a drummer-boy of 14 would now be 98 years old!

### "Brotherly Love."

By a Nurse.

A glimpse into the receiving-room of a London hospital. Time, about half-past nine p.m.—"Promise me you won't hurt her, Sister." These words were said to me in the shrillest, yet most entreating, of voices one Saturday night as I stood by the table in the receiving-room. I looked round, and at the open door stood the true type of our London street arab. A boy of about eight years of age, dirty, without shoes or stockings, and in very ragged clothes, holding by the hand a tiny child of about two and a-half years. The child had a deep wound on the forehead nearly one and a-half inches in length, caused by a fall on the curbstone. I saw at a glance that the wound was deep, and would require surgical treatment, so, sending the boy to wait in the out-patients' room, I took the frightened little sister with me, and placed her on the couch ready for the house surgeon. The child was very good whilst the wound was stitched, and did not utter a sound. The doctor was so pleased with her that he gave her a penny, and called her a "little brick." The wound stitched and bandaged, I took her myself to her brother, and told him the doctor had given her a penny. The boy's delight knew no bounds, and, folding the child in his diminutive arms, he kissed her, and then in his shrill, piercing voice called out, "Big brother go buy you sweeties, darlin'! Big brother go buy you sweeties!" And with these words "big brother" and baby sister went off happy in the expectation of the pennyworth of sweeties which would be bought at the late hour of ten p.m. So Saturday night ended happily for these two poor waifs, probably it was the happiest night they had had all that week.

